# Multiple imputation of missing data in multilevel ecological momentary assessments: an example using smoking cessation study data

**DOI:** 10.3389/fdgth.2023.1099517

**Published:** 2023-11-10

**Authors:** Linying Ji, Yanling Li, Lindsey N. Potter, Cho Y. Lam, Inbal Nahum-Shani, David W. Wetter, Sy-Miin Chow

**Affiliations:** ^1^ Department of Biobehavioral Health, The Pennsylvania State University, University Park, PA, United States; ^2^ Department of Psychology, Montana State University, Bozeman, MT, United States; ^3^ Department of Human Development and Family Studies, The Pennsylvania State University, University Park, PA, United States; ^4^ Center for Health Outcomes and Population Equity, Huntsman Cancer Institute, and Intermountain Healthcare Department of Population Health Sciences, University of Utah, Salt Lake City, UT, United States; ^5^ Data-Science for Dynamic Decision-Making Center (d3c), Institute for Social Research, University of Michigan, Ann Arbor, MI, United States

**Keywords:** multilevel data, multilevel multiple imputation, non-ignorable missing data, ecological momentary assessments, multilevel Bayesian vector autoregressive model

## Abstract

Advances in digital technology have greatly increased the ease of collecting intensive longitudinal data (ILD) such as ecological momentary assessments (EMAs) in studies of behavior changes. Such data are typically multilevel (e.g., with repeated measures nested within individuals), and are inevitably characterized by some degrees of missingness. Previous studies have validated the utility of multiple imputation as a way to handle missing observations in ILD when the imputation model is properly specified to reflect time dependencies. In this study, we illustrate the importance of proper accommodation of multilevel ILD structures in performing multiple imputations, and compare the performance of a multilevel multiple imputation (multilevel MI) approach relative to other approaches that do not account for such structures in a Monte Carlo simulation study. Empirical EMA data from a tobacco cessation study are used to demonstrate the utility of the multilevel MI approach, and the implications of separating participant- and study-initiated EMAs in evaluating individuals’ affective dynamics and urge.

## 1. Introduction

Advances in data collection technology and data modeling techniques offer many opportunities for leveraging intensive longitudinal data (ILD) to better understand the evolution of health processes over time. The introduction of ecological momentary assessment (EMA) in studies of tobacco and substance use (1) has led to a dramatic increase in the number of studies that utilize EMAs. EMA studies are powerful in elucidating the everyday, real world processes that affect individuals’ risk for tobacco use and maintaining abstinence during a quit attempt (2–7). However, EMA studies are also especially susceptible to missingness issues related to noncompliance, attrition over time (8, 9), and technological glitches (e.g. a completed EMA may not be saved or uploaded to the cloud if the smart phone crashed after a participant completed an EMA).

Despite recent EMA methodologies that consider how burden and disengagement may influence missing data (e.g., (10–12)), limited extant work exists to help illuminate the extent to which use of distinct missing data handling techniques would impact results and conclusions in analyses of EMA data, which are typically multilevel (e.g., with repeated occasions nested within individuals). Some of the previous work focused, instead, on studying how modern missing data techniques, such as multiple imputation (MI) and full information maximum likelihood estimation (FIML), can be adapted to the characteristics of intensive longitudinal data (for a review, see (13)). Attempts have also been made to apply missing data handling techniques to multilevel cross-sectional data (e.g., with linear regression models; for a review, see (14, 15)). However, it is still not clear how to adapt missing data handling approaches to multilevel ILD data.

ILD arising from EMA studies are, by nature, clustered, with measurement occasions nested within persons. Prior studies showed between-person heterogeneity in behavioral and psychological dynamics (e.g. changes of urge levels, lapses, and motivation to quit) during tobacco cessation studies. For instance, lower socioeconomic status (SES) smokers were more likely to experience smoking lapses (16), and neurotic participants tended to report higher levels of negative affect (17, 18), which may, in turn, have implications for intervention outcomes. Given the importance of modeling such between-person differences in intraindividual dynamics, it is crucial to account for data clustering structure in ILD analysis. One of the popular ways to analyze such clustered data is to apply multilevel modeling techniques to dynamic models (19, 20). Examples of such models include multilevel vector autoregressive models (MVAR) and related variations (20, 21), including MVAR variations that have become well-known in the structural equation modeling literature as dynamic structural equation models (22–24). One purpose of this paper is to illustrate the application of missing data handling techniques with MVAR model.

### 1.1. Missing data handling methods for longitudinal data

Understanding the mechanisms through which the data became unobserved is important for deciding the appropriate missing data handling approach. Based on Rubin’s classification, data may be missing under one of three possible missing data mechanisms: missing completely at random (MCAR), missing at random (MAR), and missing not at random (MNAR) (25). Let Y represents a matrix of variables observed from n participants, with some missing observations. Suppose z is a fully observed variable, which is not of substantive modeling interest but may be related to the causes of missing observations in Y, or the values of the missing observations in Y. z is often referred to as auxiliary variable. R represents the missing data indicator matrix associated with each observation of Y. Each element of R, denoted as r, takes on the value 0 if the corresponding y is observed, and 1 if y is missing. According to Rubin’s (25) definition, when the probability of r=1 does not depend on any variables of research interests, either observed or missing, the missing-data mechanism is called MCAR. In the cases of MAR, the causes of missing data in Y depend on the observed data, z, or observed covariates in the analysis model, but not on Y itself. For MNAR conditions, the cause of missingness is unobserved. The probability of Y being missing depends on values of Y itself, or on some other unmeasured variables which are related to Y. Thoemmes and Mohan provided a graphical illustration of missing data mechanisms in their work (26). Thus, in the context of EMAs in tobacco cessation studies, if instances of participant-initiated assessments are systematically higher or lower in values of the study variables compared to other instances on which the participants are simply prompted to respond at fixed or random intervals, analyzing only one set of assessments without regard to other sets of assessments could yield data that are MNAR.

Failure to account for data missingness can lead to much more severe consequences in longitudinal data analysis. In dynamic models, dependent variables (DV: Y) serve not only as the outcome variables but also as predictors of later time points. When fitting a dynamic model, missing data methods that ignore missing data and perform model fitting procedures only with fully observed occasions (e.g., listwise deletion) would produce biased parameter estimates and low power (27), even under MCAR and MAR conditions. One important reason that leads to the biased estimates is dropping observations with missing variables inevitably alters the true time intervals between observations, which would violate the equal-interval assumption of many time series models that relate the observations at time t to those from some discrete time steps earlier (t−1, t−2, etc.).

Modern missing data methods usually involve imputation implicitly or explicitly, where missing values were filled in with predicted scores based on specified models (28). Examples of implicit imputation were FIML and Data Augmentation (29), both of which simultaneous handle missing data and statistical modeling, where model-based-imputed values were generated during the estimation process. MI, an explicit imputation method proposed by Rubin (30) has been widely applied in cross-sectional survey data (31), and found to be effective in handling most of missing data problems (31–34). MI methods have also been extended to handle missing data problems in longitudinal panel studies (35, 36), longitudinal clinical trials (37, 38), and intensive longitudinal data models (13). MI is a two-step, data-based approach that handles the missing data in one step, and then estimates the full-data model in a separate step (39). Specifically, MI procedures first generate multiple sets (the number of imputations is denoted as m, where m>1) of possible values of missing observations, using observed variables based on predefined imputation model. The m sets of imputed values then substitute the missing values in the original dataset, producing m versions of plausible imputed data sets. Each imputed dataset is then used in model fitting procedures as if all data were observed, resulting in m sets of parameter estimates. Combining the m sets of parameter estimates, one set of pooled parameter estimates, $\bar{Q}$, can be obtained. Rubin’s (30) rules in pooling parameter estimates specify that the pooled parameter point estimates are simply the average of the parameter estimates over the m sets of parameter estimates from each model fitting procedure. For pooled standard error (SE) estimates, both within—imputation variability and between—imputation variability need to be accounted for.

**Figure 1:**
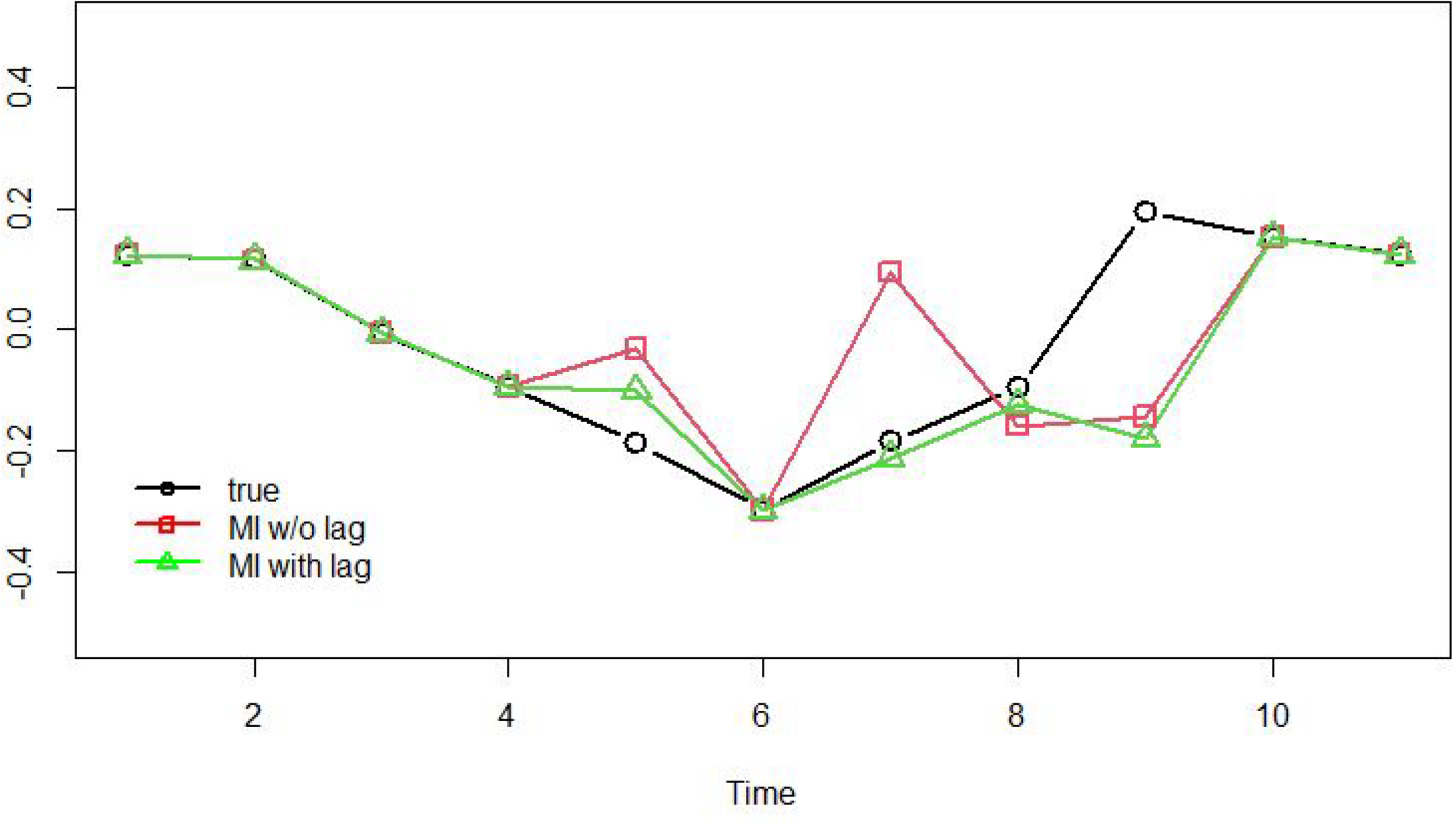
This plot compares multiple imputation with and without lagged variable against true data under the context of autoregressive model of order 1, 11 time points. The black circles represent true data generated using an autoregressive model of order 1, where time-points 5, 7, 8, 9 were set to be missing. The red squares represent average multiply imputed data points with m=5 when the imputation model does not include lagged variable. The green triangles represent average multiply imputed data points with m=5 when the imputation model includes lagged variable.

A simulation study by Ji et al. (13) showed that using imputation models with lagged variables when the true model was a VAR model improved both parameters’ point estimates and SE estimates compared with approaches that did not utilize the lagged information effectively. To illustrate the importance of including lagged variable, we present a comparison of imputed data points for missing observations using simulated data with an autoregressive model of order 1 (see, Figure 1). In particular, when lagged variable (i.e., observation from the previous time point) was included in the multiple imputation model, the mean imputed observations at time point 5 and 7 were less biased than performing multiple imputation without lagged variable. They proposed a partial MI approach in which missing data in covariate variables are imputed with MI procedures, whereas missing data in DVs are retained in the dataset and handled using the FIML approach. Simulation study results showed that this partial MI approach produced the best estimation results, especially for point estimates of time-series parameters that convey the dynamics of the system over time (e.g., autoregression and cross-regression parameters, as defined explicitly in a later section).

In recent years, research efforts have been dedicated toward extending standard MI procedures to multilevel models. Previous research has shown that failing to account for this nested structure of the data in the MI procedures may lead to different variance and covariance properties of the imputed data, as compared with the actual data (14). As a result, substantive model estimation results may be biased. The extent to which the parameters are biased depends on many factors, including whether the parameters are within- or between- level parameters, the intra-class correlation (ICC) of the data set (i.e., the amount of variability accounted for by systematic between-person differences in means (referred to as random intercepts in MVAR models) relative to the total variability of the data). Simulation studies found that applying single-level MI to clustered data resulted in a biased estimation of between-group and within-group regression coefficients independent of sample size (40). However, all these properties are observed with cross-sectional data with linear models. Even though, the use of MI for longitudinal panel data in the context of linear regression and linear mixed-effects model were explored and evaluated (41, 42), the consequences of using single-level MI procedures with intensive longitidinal clustered data when fitting dynamic models involving time-lagged relationships are not well understood.

### 1.2. Study objectives

The purpose of this article is twofold. First, we highlight the importance of accounting for the multilevel structure of ILD from EMA studies, particularly when implementing missing data handling procedures in the context of applying multilevel model to multi-subject data. To illustrate this point, we used simulated data sets designed to mirror the characteristics of ILD from EMA studies to compare estimation results when treating data from multiple individuals as single level data versus using a multilevel multiple imputation (multilevel MI) procedure. Second, we illustrate one application of multilevel MI using empirical data from a longitudinal smoking cessation study. In this illustration, we evaluate the tenability and sensitivity of conclusions concerning bi-directional dynamic relations between negative affect and urge. Specifically, by comparing estimation results with different missing data handling approaches, including multilevel MI, single-level MI, Bayesian_FIML, and listwise deletion, we aimed to evaluate whether and to what extent conclusions concerning the lead-lag relations between negative affect and urge, interindividual differences, and correlates with time-varying risk processes vary under different missing data handling techniques. Note that even though we refer to individual as the unit for analysis throughout this paper, the illustrated approaches may also apply to a couple/dyad, a family, or other units of analysis.

## 2. The general MVAR model

The MVAR model has two components: the within-level model, and the between-level model. For the within-level model, we considered a bivariate VAR(1) model for the dynamic process, with $y_{1,i,t}$ and $y_{2,i,t}$ influencing themselves as well as each other at the subsequent time point. This model essentially captures the interdependence between the two change processes, $y_{1,i,t}$ and $y_{2,i,t}$, through how much the values of these processes from an earlier time point, t−1, affect their current values at time t. Person-specific intercepts are incorporated to represent between-person differences in baselines for these change processes. These baseline values serve as anchor points which the processes of interest fluctuate around and represent the average levels of these processes within an individual. In other words, a high (low) value on a DV is high relative to each person’s usual baseline, not necessarily high (low) relative to other individuals. The model also includes two time-varying COVs, $x_{1,i,t}$ and $x_{2,i,t}$, as part of the dynamic model. These COVs are measured person- and time-specific independent variables thought to affect the values $y_{1,i,t}$ and $y_{2,i,t}$, but the change processes that govern them are typically not of substantive interest to the researchers.

The within-level model for MVAR was expressed as:

(1) $$y_{1,i,t}-\mu_{1,i}=ar_{1,i}*(y_{1,i,t-1}-\mu_{1,i})+cr_{1,i}*(y_{2,i,t-1}-\mu_{2,i})+c_{1}*x_{1,i,t}+d_{1}*x_{2,i,t}+\zeta_{1,i,t}$$

(2) $$y_{2,i,t}-\mu_{2,i}=ar_{2,i}*(y_{2,i,t-1}-\mu_{2,i})+cr_{2,i}*(y_{1,i,t-1}-\mu_{1,i})+c_{2}*x_{1,i,t}+d_{2}*x_{2,i,t}+\zeta_{2,i,t}$$

(3) $$\left[\begin{array}{c} \zeta_{1,i,t}\\ \zeta_{2,i,t} \end{array}\right]∼N\left(\left[\begin{array}{c} 0\\ 0 \end{array}\right],\Sigma =\left[\begin{array}{c} \sigma_{1}^{2}\;r_{\sigma_{1}\sigma_{2}}\\ r_{\sigma_{1}\sigma_{2}}\;\sigma_{2}^{2} \end{array}\right]\right),$$

The between-level model for MVAR was expressed as:

(4) $$g(\theta_{i})=BX_{i}+\nu_{i},\;\nu_{i}∼N(0,\Delta ).$$

where $y_{1,i,t}$ and $y_{2,i,t}$ are the two DVs observed for the person i at time point t; $\mu_{1,i}$ and $\mu_{2,i}$ are person-specific intercepts. In tobacco cessation study, DVs could be participants’ urge to smoke and participants’ negative affect, for example. $y_{i,t}-\mu_{i}$ represent the amounts of deviation in cravings and negative affect from each person’s respective baseline ($\mu_{i}$) at time t. $ar_{1,i}$ and $ar_{2,i}$ represent person-specific auto-regression (AR) parameters, capturing how much the amount of deviations at time t are related to the person’s deviations from the baselines at the previous time point. $cr_{1,i}$ and $cr_{2,i}$ represent person-specific cross-regression (CR) parameters. At the between-level model, $\theta_{i}$ is a matrix that consists of all person-specific parameters that appear in Equations 1, 2, and 3. Variables $X_{i}$ included exogenous variables that explain some of the between-person differences in the person-specific parameters, and B is the matrix of regression coefficients (or fixed effects) at the between-level model. $\nu_{i}$ represent random effects with distributions of zero means and the random effect covaraince matrix Δ.

We fit the MVAR model using the R package “rjags,” with the default MCMC algorithms (43, 44). The MCMC algorithms perform iterative sampling to get posterior distributions for each parameters based on approximate conditional distributions. During the sampling procedures, adaptation is performed by the algorithms to automatically tune the parameters. After that, iterations during the initialization stage are discarded as burn-in. Usually, longer number of iterations may produce higher quality results. When autocorrelation is observed with the posterior, we use thinning so that the algorithm only retained the samples at every nth iteration. We assessed the convergence of the procedures using $\hat{R}$ (i.e., threshold <1.1), which is the ratio of the overall variance to the within-chain variance of posterior samples across chains (45). In addition, we also inspected the effective sample size (ESS) to ensure that the number of independent posterior draws was greater than the minimum threshold of 300 for all parameters (45).

The choice of prior distribution plays an vital role in the estimation of Bayesian models. Weakly informative priors were used based on prior knowledge of the possible range of the parameters. Specifically, for the MVAR(1) model with random intercepts and random-VAR-related parameters, the priors for the between-level model parameters were set to a normal distribution with means of 0 and variances of 100. The error variances of the between-level model were set to uniform distribution with lower bound at 0 and upper bound at 100. When VAR-related parameters were fixed, instead of random, I used normal priors with mean of 0 and variances of 10. For covariate parameters ($c_{1},c_{2},d_{1},d_{2}$), normal priors with means of 0 and variances of 1 were used. Given the permissible values of VAR-related parameters for stationary time-series models, this set-up of priors was more diffuse than the parameter values we would expect for stationary MVAR(1) models, which typically fell within the range of [−1.5,1.5] (46, 47). For the process noises variance-covariance (referred to as “var-cov” hereafter) parameters, variances parameters had an uniform prior between 0 and 100, and the correlation parameter had an uniform prior constrained between the values of −1 and 1.

Fitting models in JAGS yields posterior distributions for each model parameter, from which we can obtain point and standard error estimates, and credible intervals (similar to “confidence intervals” in the frequentist framework) by calculating the distributions’ means/medians, standard deviations, and quantiles respectively.

## 3. Simulation study: consequences of ignoring nested data structures in performing MI

### 3.1. Simulation designs

What are the consequences of ignoring multilevel or nested (e.g., repeated measures nested within days and/or within individuals) data structures in performing MI procedures? Previous studies showed that different missing data handling procedures performed differently when the true data-generation model and the analysis model were random-intercept-only multilevel models and multilevel models with random slope coefficients for cross-sectional data (40, 48). To investigate if the findings still hold with multilevel dynamic models, we demonstrate the consequences of inadvertently performing MI procedures on data that do have a nested structure in the context of a dynamic model known as a random-intercept-only MVAR model (details forthcoming) under scenarios with relatively high compared to low magnitudes of interindividual differences in intercepts (i.e., high vs. low ICC). We assume that the data are MAR. We also compared the performance of the different MI approaches to a Bayesian FIML (BFIML) method, a broadly accepted method to handle missing data that has been shown to work well under MAR conditions (49). This BFIML approach is similar to the data augmentation approach implemented in Bayesian factor analysis models (50). With the BFIML model, model-implied observed and/or latent variable values are used as the “imputed” scores for missing observations in DVs, and missing data in covariates (COVs) are handled through specification of appropriate models and distributional assumptions for the COVs. The underlying assumption for this approach is that all missing data follow the MAR or MCAR mechanism. For each simulation condition, we ran 100 Monte Carlo replications.

Complete-data sets were generated based on a random-intercept-only MVAR model as described in the previous section (See Equations 1–4). In this simulation, we considered a random-intercept only MVAR model, therefore the VAR-related parameters (i.e., $ar_{1,i}$, $ar_{2,i}$, $cr_{1,i}$, and $cr_{2,i}$) are fixed at the same value for all persons. Later in the empirical example, we included both random-intercepts and random VAR parameters in the model. The effects of the time-varying COVs, $x_{1,i,t}$ and $x_{2,i,t}$, on the process are estimated by parameters $c_{1}$, $c_{2}$, $d_{1}$, and $d_{2}$. The time-varying COVs are generated following an AR(1) model similar to the DV processes. The process noises $\zeta_{i,t}$ conforms to a multivariate normal distribution with a zero mean vector and a covariance matrix Σ, in which $\sigma_{1}^{2}$ and $\sigma_{2}^{2}$ represent variances of the process noises and $r_{\sigma_{1}\sigma_{2}}$ represent the covariance. At the between-level model, all person-specific parameters in Equation 1 are regressed on a time-invariant person specific COV ($x_{3,i}$). The MVAR may be considered as a special case of dynamic structural equation models (22, 23), or an extension of the actor-partner interdependence model (51) widely used in the context of dyadic data analysis. Values for the model parameters were selected to reflect the parameter values we often observe with dynamic models’ applications in empirical studies (e.g., (52–54)). True values for the VAR parameters were set at .4 and .3 for ars and −.3 and −.2 for crs. Parameter values for the effects of the time-varying covariates on the process, $c_{1}$, $c_{2}$, $d_{1}$, and $d_{2}$, were .3, .3, −.5, and −.4, respectively. The process noises variances, $\sigma_{1}^{2}$ and $\sigma_{2}^{2}$, were 1 and the covariance was set at .3.

To simulate MAR missing condition, the probability of having a missing value in the DVs and $x_{1,i,t}$ only depended on the observed time varying covariate $x_{2,i,t}$. (see Equations 5 and 6, where $\psi_{0}$s represent intercepts of the missing data model, $x_{2}$ represents the observed time varying covariate without missing data, and $\psi_{1}$ and $\psi_{x_{2}}$ represent coefficients related to the time varying covariate in the missing data model.) Values of missing data model parameters were chosen to achieve the target percentages of missingness of 30%, moderate level of missingness for EMA studies. A comparison of two missing data handling procedures were presented in this illustration: (1) single-level MI, ignoring the multilevel structure (single-level MI) and (2) multilevel MI. Both MI missing data handling methods included two steps. First, missing observations in DVs and the time-varying covariate were imputed using different MI methods, including single-level MI and multilevel MI, generating five sets of possible values for the missing observations. Imputation models included all variables involved in the MVAR(1) model, as well as auxiliary variables that were helpful for the imputation procedures but not modeled in the MVAR(1) model. Specifically, variables included in the imputation model are the two DVs ($y_{1,i,t}$ and $y_{2,i,t}$), the lagged DVs ($y_{1,i,t-1}$ and $y_{2,i,t-1}$), the time varying covariates ($x_{1,i,t}$ and $x_{2,i,t}$), the level 2 predictor, and the variables used to generate the covariates as auxiliary variables. Those imputed values were then filled in to the dataset with missingness, and as a result, five complete datasets were created. The second step was the Bayesian MVAR(1) model fitting procedure with the complete data set, which was performed using the statistical software “Just Another Gibbs Sampler” (JAGS; (43, 44)). After this step, we obtained five sets of parameter estimates, as well as posterior distributions of all parameters of interest, corresponding to the five imputed data sets. We used the posterior distributions of the parameter estimates from the five model fitting procedures to calculate summary statistics such as mean, standard deviation, and credible intervals. This approach took into consideration the variability of parameter estimates both within each imputation and across different imputations.^1^

(5) $$P(r_{DV_{i,t}}=1|x_{2,i,t})={\mathrm{logit}}^{-1}(\psi_{0DV}+\psi_{1}x_{2,i,t})$$

(6) $$P(r_{x_{1,i,t}}=1|x_{2,i,t})={\mathrm{logit}}^{-1}(\psi_{0x_{1}}+\psi_{x_{2}}x_{2,i,t})$$

The performances of the different missing data handling approaches were evaluated under different sample size conditions (i.e., combinations of small/large N and small/large T) and different intraclass correlation coefficient (ICC) levels (i.e., the proportion of the total variance that is explained by the between-person differences (23)). The performance of the different missing data procedures were assessed using the following criteria. The accuracy of point estimates of the model parameters were evaluated using both average bias (Equation 7), average relative bias (Equation 8) and average root-mean-squared error (RMSE, Equation 9) across MC runs, which were calculated as:

(7) $$\text{b}ias(\theta )=\frac{1}{H}\sum_{h=1}^{H}({\hat{\theta }}_{h}-\theta )$$

(8) $$\text{r}elative\;bias(\theta )=\frac{1}{H}\sum_{h=1}^{H}\left(\frac{{\hat{\;\theta }}_{h}-\theta }{\theta }\right)$$

(9) $$\text{R}MSE(\theta )=\sqrt{\frac{1}{H}\sum_{h=1}^{H}({\hat{\;\theta }}_{h}}-\theta {)}^{2},$$

where H represented the total number of MC runs, ${\hat{\;\theta }}_{h}\;$ represented the estimated parameter value for the hth MC run, and θ represented the true parameter value used in the data generation procedure. Compared with bias and relative bias, RMSE provided additional information on the variability of the estimates.

The quality of the SE estimates was assessed with differences between the average SE across MC runs and the empirical MC standard deviations of the parameter estimates (dSD, Equation 10). The dSD was calculated with:

(10) $$\text{d}SD(\theta )=\frac{1}{H}\sum_{h=1}^{H}{\text{S}E}_{h}(\;\hat{\;\;\theta }\;)-\sqrt{\frac{1}{H-1}\sum_{h=1}^{H}(\;{\hat{\;\theta }}_{h}-\;\bar{\;\;\theta }\;{)}^{2}},$$

where $\bar{\;\;\theta }=\frac{1}{H}\sum_{h=1}^{H}{\hat{\;\theta }}_{h}$, ${\text{S}E}_{h}(\hat{\theta })$ represented SE estimates of parameter θ for the hth MC run.

The R package, mice (55), was used for both single-level MI and multilevel MI approaches. Detailed procedures for the single-level MI method followed the steps described in Ji et al. (13). For the multilevel MI method, we followed the recommended steps detailed in vignette 5 of the mice r package (56). In the imputation procedure, participant IDs were included in the model, allowing for between-person heterogeneity in the imputation procedures.

### 3.2. Simulation results

To facilitate the presentation of the simulation results, parameters of the same type that showed the same patterns across different conditions with different approaches were grouped together, and averaged results were presented. We refer to the AR ($ar_{1}$ and $ar_{2}$) and CR ($cr_{1}$ and $cr_{2}$) parameters as VAR parameters; the parameters involved in the between-level model for the random intercepts (i.e., fixed-effects and random-effect variances) as random-intercept parameters; the fixed effects for the time-varying COVs, $c_{1}$, $c_{2}$, $d_{1}$, and $d_{2}$, as COVs parameters.

**Figure 2:**
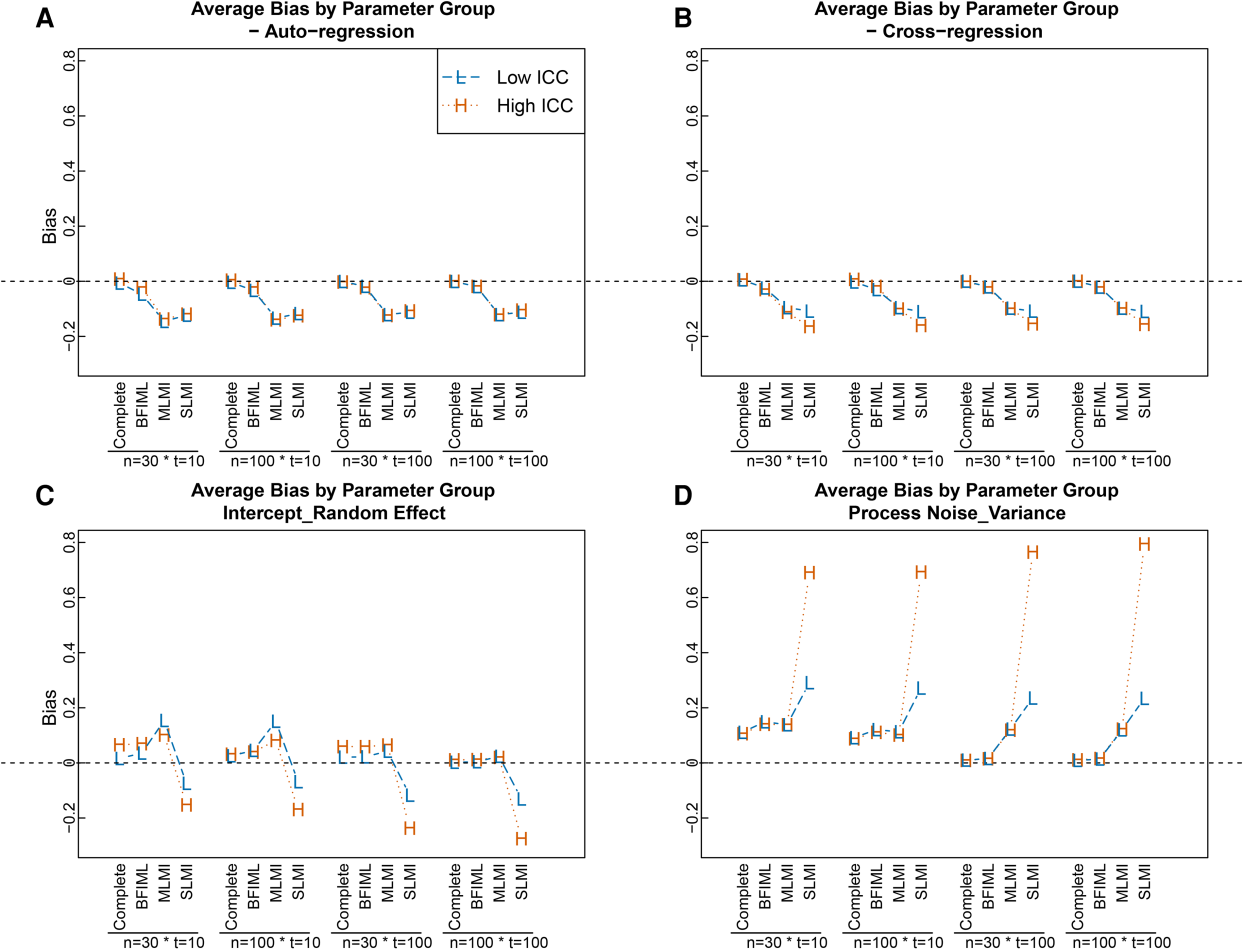
Average bias in parameter estimates in different modeling approaches and with different sample sizes, grouped by parameter category. Bias was calculated by: $\text{b}ias(\theta )=\frac{1}{H}\sum_{h=1}^{H}({\hat{\;\theta }}_{h}-\theta )$. Parameters with estimation biases close to zero across all conditions, including covariate parameters, process noise covariance, and fixed effects for random intercepts, were not included in the plot to ease presentation. Complete = Results from fitting model with complete data set; BFIML = Results with Bayesian full-information maximum likelihood method; MLMI = Results with multilevel MI; SLMI = Results with single-level MI.

We observed that when the multilevel structure of the data was not accounted for in the imputation procedure, estimation results were biased both in terms of point estimates and SE estimates for most of the parameters across all sample size conditions. Among all the parameters in the hypothesized MVAR model, single-level MI for the multilevel data led to the most biased estimates for the random intercepts variances at the between-level and the process noise variance at the within-level. Specifically, as Figures 2C,D indicated, when single-level MI were used, process noise variances for the within-level VAR model were over-estimated, whereas the random intercepts variances were under-estimated across all sample size conditions. The relative bias for the parameter estimates were also higher with single-level MI when compared with multi-level MI. For instance, the mean relative bias for process noise variance parameters under the high ICC and large sample size condition with single-level MI was 0.47, compared to −0.02 when multi-level MI was used. For the intercept random effect under the same condition, relative bias with single-level MI was −0.2 and dropped to 0.01 when multi-level MI was used. Under the same simulation condition, smaller root-mean-square error (RMSEs) were observed for the parameter estimates with multi-level MI, as compared with single-level MI for intercept random effects and process noise variances. Specifically, average RMSE for process noise variance parameters with single-level MI was 0.24, compared to 0.12 with multilevel MI. For intercept random effect parameters, RMSEs with single-level MI was 0.29, compared to 0.1 with multi-level MI. This was mainly due to the fact that in the imputation procedure, variability in the intercepts was not accounted for with single-level MI. In addition, Figure 2B also shows that CR parameters were under-estimated with single-level MI under high ICC condition, but the differences were relatively small for this particular set of parameters.

**Figure 3:**
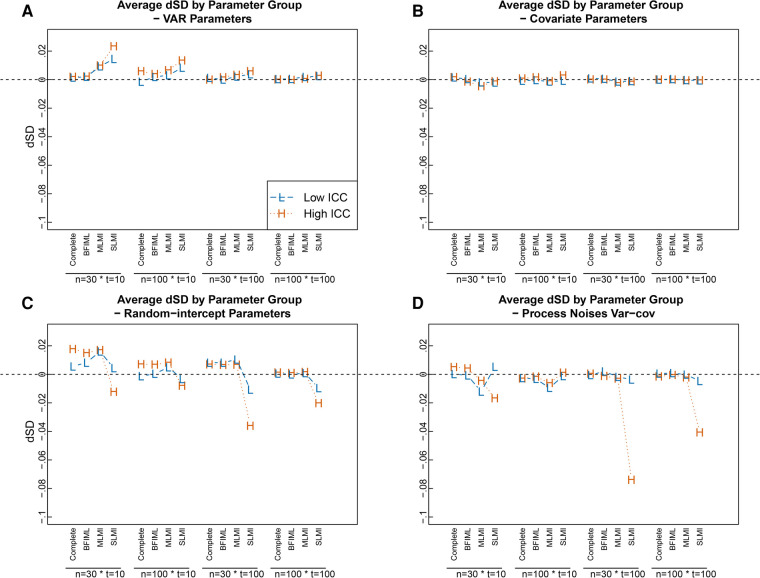
dSDs in parameter estimates in different modeling approaches and with different sample sizes, grouped by parameter category. The dSDs was caluclated with $\text{d}SD(\theta )=\frac{1}{H}\sum_{h=1}^{H}{\text{S}E}_{h}(\hat{\;\theta })-\sqrt{\frac{1}{H-1}\sum_{h=1}^{H}({\hat{\theta }}_{h}-\bar{\;\theta }{)}^{2}}$. Random-intercept parameters here include fixed-effects parameters and random-effect variances. Complete = Results from fitting model with complete data set; BFIML = Results with Bayesian full-information maximum likelihood method; MLMI = Results with multilevel MI, pooled using posteriors; SLMI = Results with single-level MI, pooled using posteriors.

Similarly, the results for SE estimates when single-level MI was used were worse for random-intercept parameters and process noise var-cov parameters compared with other approaches. When compared with the MC empirical SEs of the parameter estimates, pooled SE estimates using single-level MI tended to be smaller for random-intercept parameters and process noise var-cov parameters, especially with large sample sizes (i.e., T=100) and high ICC level (see Figures 3C,D). In addition, pooled SEs were over estimated for VAR parameters when sample sizes were smaller (i.e., T=10) (see Figure 3A). The difference of SE estimates for covariate parameters were similar across missing data handling approaches most due to no between person differences were involved for time-varying covariate effects (see Figure 3B).

The BFIML approach performed reasonably well for all simulation conditions, both in terms of parameter point estimates and standard error estimates, in this simulation study. This was illustrated by the very small bias and dSD for all parameters in Figures 1 and 2. The exceptionally good performance of BFIML in this simulation was mainly due to the fact that both the dependent variable model and covariate models were correctly specified according to the data generation model. In addition, all missing observations were missing at random, which aligns with the BFIML missing data assumption, and thus no additional missing data model specification is needed (see (13) for Bayesian selection models). However, with the multi-level MI approach, imputation model was generally specified to include all available information that was considered to be helpful in predicting the missing observations. The imputation model did not follow the data generation model in terms of the functional form, but instead, represented a more general imputation model. Future research shall exam the performance of BFIML approch with non-ignorable missing data, and the robustness of the approach with different levels of model miss-specification, such as miss-specified dependent variable model, covariates model, and missing data model.

The simulation results suggested that for most within-level model parameters, a relatively small sample size with T=10 and N=30 was good enough to get reasonably good parameter point estimates with random-intercept-only MVAR(1) model, even with 30% of missing data, when the missingness was properly handled (i.e., with multilevel MI or BFIML). However, to get better estimates for process noise var-cov parameters, larger T would be helpful (see Figure 2D). In terms of SE estimates, a larger T×N combination condition produced better SE estimates for all parameters (see Figure 3) when missing data were properly handled. However, when improper missing data handling approach was used (i.e., single-level MI), larger sample size did not improve SE estimates for random-intercept parameters and Process Noises Variance-covariances, especially when ICCs were high.

The point estimates and SE estimates of covariate parameters were reasonably good with single-level MI. This was because we did not have random effects for the covariate parameters in the data generation model. In other words, when we generate the time-varying covariates, we assumed no random intercept and no random effect for the AR(1) parameters. As a result, the time-varying covariates only have within-person variations at level 1 and do not have individual differences in the within-person parameters at level 2. Thus using single-level MI was appropriate for imputing the missing covariate in this simulation scenario.

## 4. Empirical example: bidirectional association between urge and negative affect during smoking cessation intervention

We demonstrate the utility of multilevel MI procedures and the impact of different missing data handling procedures on estimation results using a tobacco cessation EMA study. ILD from this study was used to investigate (1) how smoking urge and negative affect relate to each other across time as well as to other factors including cigarette availability and lapse; (2) how participants differ in their baseline levels of urge and negative affect, as well as the bi-directional time-lagged relationships between urge and negative affect; and (3) how demographic characteristics were associated with between-person differences in baseline levels of urge and negative affect.

### 4.1. Data descriptions

#### 4.1.1. Participants

Data used in this study were collected in a National Institute on Drug Abuse–funded longitudinal study on factors (e.g., abstinence self-efficacy, positive affect, negative affect, stress, urge) influencing smoking cessation among 424 participants who were recruited between 2005 and 2007 from the Houston, Texas area. Participants were at least 21 years old at the time of enrollment, smoked at least five cigarettes per day on average for the last year, and were motivated to quit within the next month. Four random EMAs were scheduled to be delivered each day during typical waking hours. Participants were also instructed to self-initiate nonrandom EMAs when they were about to smoke (smoking EMAs), experienced an urge to smoke (urge EMAs), or had already slipped (slip EMAs). Among the 424 participants who enrolled in the study, 43 did not complete any EMAs and were excluded. Data for the current study included both random and nonrandom assessments during the 28 days of postquit monitoring. More details about the study can be found in previous studies (see, e.g., (16, 57)).

Since the time intervals between EMAs varied within individuals over time, we aggregated the raw data to be equally spaced so that a discrete-time model could be fitted. As in previous studies (52, 58), we aggregated EMA data into four blocks (i.e., 12a.m.–6a.m., 6a.m.–12p.m., 12p.m.–6p.m., 6p.m.–12a.m.) per day to represent the sleeping, morning, afternoon, and night periods. For instance, the negative affect data (either from random EMAs or self-initiated EMAs) collected during 6p.m.–12a.m. were averaged to represent their levels of negative affect during the night period. Such aggregation was implemented on both random and self-initiated EMAs. Based on the aggregated data, we excluded participants who had less than 8 total measurement occasions, yielding a sample size of 366, with number of days ranging from 4 to 28 and number of time points ranging from 14 to 112.

#### 4.1.2. Measures

The dependent variables and time-varying COVs were measured by random EMAs during the 28-day postquit period, except for smoking lapse which was measured by random, urge and slip EMAs in the same time period. We only considered random EMA observations for the dependent variables to be in correspondence with prior published work using the same data set (57). The time-invariant COVs were measured at baseline; and the auxiliary variables were measured by both random and self-initiated EMAs as detailed below.

##### 4.1.2.1. Dependent variables (DVs)

**Urge** was measured by the mean of 3 items, “I have an urge to smoke,” “I really want to smoke,” and “I need a cigarette.” which were rated on a scale of 1 (strongly disagree) to 5 (strongly agree).

**Negative affect** was measured by the mean of 5 items asking if the respondent felt bored, sad, angry, anxious, or restless, which were rated on a scale of 1 (strongly disagree) to 5 (strongly agree).

Note that in the context of MVAR models, linear or systematic trends in the DVs have to be removed prior to model fitting in order to meet the stationarity assumption of the model and avoid spurious effects (see, e.g., (46)). We removed the linear trends in the DVs by first regressing urge and negative affect on measurement occasions, respectively, to obtain their residuals, and then added their person-specific means back to the residuals to obtain the final scores for urge and negative affect to be used in model fitting.

##### 4.1.2.2. Time-varying covariates

***Smoking lapse*** was measured by both random and self-initiated EMAs. For random and urge EMAs, participants responded to a single item asking if they had smoked any cigarettes that they had not already recorded in the computer. Those indicating lapse responded to two additional items, “How many cigarettes did you smoke that you did not record?” and “How long ago did you smoke the most recent cigarette that you did not record?”. For slip EMAs, participants responded to two items, “How many cigarettes did you smoke during this slip?” and “How long ago did you smoke your last cigarette?”

Both random and self-initiated smoking lapse items assessed the time that a lapse occurred with 7 response options ranging from “0–15 min” to “8 h or more”. Time of lapse was measured by subtracting the midpoint of the response option interval (e.g., 0--15min=7.5min) from the time stamp of the current EMA. If at a particular measurement point, participants reported that they smoked the most recent cigarette 8 h ago or more, their responses to the number of cigarettes would be set to missing because the specific time of lapse could not be identified. Note that it could be the case that the time of lapse was before the previous EMA—that is, the reported smoking time was before a previous EMA. For these cases, we took the adjusted time stamp to be the time between the current and previous EMA, which was calculated by subtracting the midpoint between the previous and current EMA from the time stamp of the current EMA.

Based on these adjusted time stamps, the smoking lapse in each block was then calculated as the sum of cigarettes reported in random, urge, and slip EMAs that occurred in this specific block.

***Cigarette availability*** was measured with the item, “Cigarettes are available to me.” which was rated on a scale of 1 (not at all available, coded as 0 in analysis to facilitate result interpretation) to 5 (easily, coded as 4 in analysis).

Based on the aggregated data where each person had 4 data points per day, the overall missing rates for both DVs and time-varying COVs were 48%, with the missing rate for each participant ranging from 21% to 90%.

##### 4.1.2.3. Time-invariant COVs

In addition to these time-varying COVs, we also considered time-invariant COVs collected at baseline and investigated their effects on random intercepts and slopes. As summarized in Table 1, the time-invariant COVs included two continuous variables, age, and number of cigarettes per day (i.e., Cigsperday), as well as categorical variables measuring gender, race/ethnicity, education, whether or not participants’ partners live with them (i.e., Partnerlive), whether or not participants’ partners smoke (i.e., PartnerSmoke), and how soon participants smoked the first cigarette after they woke up (i.e., Timetofirst). The continuous variables were scaled across persons to zero means and unit variances and the categorical variables were coded as a set of dummy variables with 1 representing males, African American, Hispanic, other races, more than high school, partner lives with you, partner smokes, and 5 min or less after waking up (see details in Table 1). In terms of missing rates, there were 3 missing records in Education, and 4 missing records in PartnerLive and PartnerSmoke.

**Table 1:** Descriptives of time-invariant covariates.

Variable	n	%	Mean	SD	Min	Max
Age			42	11	21	73
Gender						
Female	203	55				
Male*	163	45				
Race						
Caucasian	120	33				
African*	120	33				
Hispanic*	118	32				
Others*	8	2				
Education						
High school or less	148	40				
More than high school*	215	59				
PartnerLive						
No partner or partner does not live with you	209	57				
Partner lives with you*	153	42				
PartnerSmoke						
No partner or partner does not smoke	294	80				
Partner smokes*	68	19				
Timetofirst						
More than 5 s	189	52				
5 s or less*	177	48				
Cigsperday			21	10	5	80

The five columns represented sample sizes, percentages, means, standard deviations, minimums and maximums, respectively.

*indicated variables coded as 1 when constructing dummy variables.

##### 4.1.2.4. Auxiliary variables

We considered urge, negative affect, and cigarette availability measured in self-initiated EMAs as auxiliary variables in the imputation procedures. We made the modeling decision to use the self-initiated EMAs of urge and affect as auxiliary variables in the imputation model, as opposed to an integral part of the DV values in the fitted MVAR model, so that the DVs in this study are consistent with prior published work on the same data set (57).

**Figure 4:**
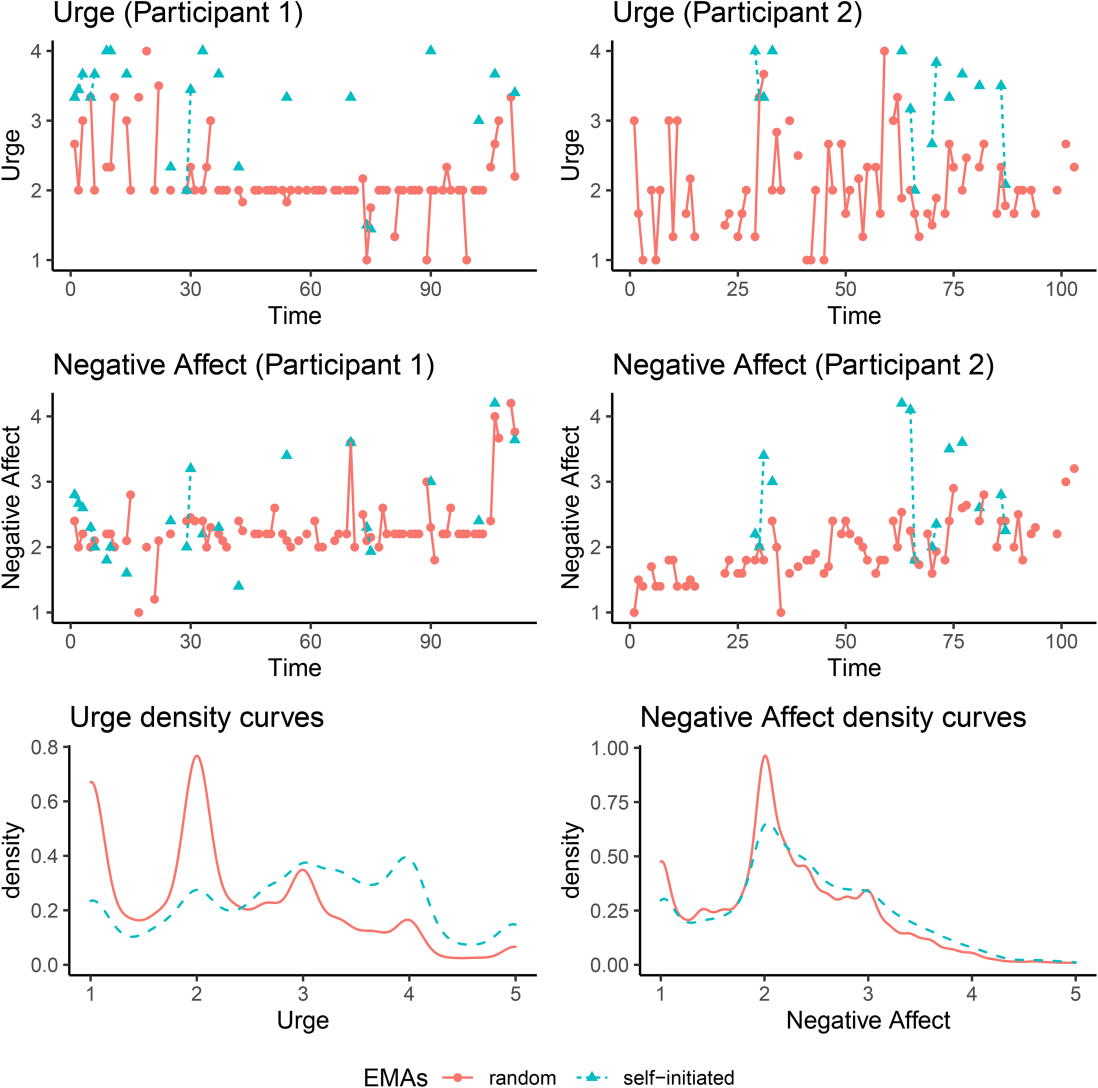
Top four panels: Trajectories of urge and negative affect in random EMAs (red solid lines) and self-initiated EMAs (blue dashed lines) for two randomly selected participants. Bottom two: Densities of urge and negative affect in random EMAs (red solid lines) and self-initiated EMAs (blue dashed lines) across all participants.

Figure 4 (top four plots) shows trajectories of urge and negative affect in random EMAs (red solid lines) and self-initiated EMAs (blue dashed lines) for two randomly selected participants, and the overall densities of these two variables across all participants (bottom two plots). It can be seen that instances of self-initiated (slip) EMAs were characterized by notably higher values of urge than instances of random EMAs. Whereas the distributions of negative affect showed only relatively minor differences during the two types of EMAs, inspection of plots of data trajectories at the individual level revealed that within participants, some of the slips EMAs were indeed characterized by distinctly higher negative affect relative to most other occasions of random EMAs, although some random EMAs were obtained near the times of the slip EMAs and thus showed largely similar negative affect values as the latter. Therefore, we included observations of self-initiated EMAs and nearby observations (i.e., lag one observations) of self-initiated EMAs in the imputation model to inform the unreported values of the random EMAs.

In addition to the above auxiliary variables, we also considered positive affect, abstinence self-efficacy, and motivation to quit measured in both random and self-initiated EMAs, all of which were measured by items rated on a scale of 1 (strongly disagree) to 5 (strongly agree). Specifically, positive affect was measured with the sum of 3 items asking if the respondent felt enthusiastic, happy, or relaxed. Abstinence self-efficacy was assessed with the item, “I am confident in my ability not to smoke.” Motivation to quit was assessed with two items, “My desire to be a nonsmoker is very strong,” and “I am extremely motivated to be smoke-free.”

In this study, 362 out of 366 participants had at least 1 measurement point from self-initiated EMAs during the postquit period. Based on the aggregated/blocked data set, participants had an average of 22 data points for auxiliary variables measured by self-initiated EMAs.

### 4.2. Data analytic plans

The empirical study model was built using a MVAR(1) model as described in Equations 1 with both random intercepts and random VAR parameters. With this model, we aimed to examine the bi-directional relationship of the two DVs across time, allowing for individual differences in those associations. We also examined how two time-varying COVs influenced the dynamic processes of the two DVs. Four different missing data handling techniques were considered in this illustration and the estimation results obtained using the four different approaches were compared. Specifically, we included two MI approaches: multilevel MI and single-level MI, a BFIML approach, and a listwise deletion approach. For the two MI approaches, the same imputation model was adopted, including all DVs, time-varying COVs, and time-invariant COVs that were part of the empirical study model. In addition, we also included auxiliary variables, such as previous time DVs and lapse, positive affect, and previous time positive affect, self-efficacy, motivation, and missing data indicators for the DVs and Time-varying COVs. Missing data indicators were included because previous simulation studies found them as important auxiliary variables (59, 60). For multilevel MI, participants’ IDs were also included as indicator of levels. For single-level MI, since different values were imputed for the time-invariant COVs, we took the average value as the imputed values. For the listwise deletion approach, all observations with missingness in time-varying COVs were dropped from the model fitting procedures. We fitted the analytic model (i.e., MVAR(1) model) using rJAGS (61).

### 4.3. Results

Model estimation results using four different approaches, including multilevel multiple imputation, single-level multiple imputation, BFIML, and listwise deletion were summarized in Table 2. With 4000 adaptation, 1000 burn-ins, 15,000 iterations and thin=2, the convergence of the MVAR models were reasonably good with ESS over 300 for parameters of interest and $\hat{R}<1.1$.

**Table 2:** Parameter estimates for empirical data.

	Multilevel multiple imputation				Single-level multiple imputation				Bayesian_FIML				Listwise deletion			
	Mean	SD	CI_Low	CI_High	Mean	SD	CI_Low	CI_High	Mean	SD	CI_Low	CI_High	Mean	SD	CI_Low	CI_High
Intercept_Urge																
Intercept	2.155	0.110	1.936	2.366	2.246	0.088	2.073	2.417	2.279	0.119	2.046	2.514	2.268	0.117	2.042	2.499
Ethnicity_African	−0.384	0.093	−0.566	−0.199	−0.087	0.075	−0.234	0.062	−0.072	0.099	−0.265	0.123	−0.081	0.100	−0.271	0.117
Partner live together	−0.373	0.089	−0.549	−0.198	−0.158	0.073	−0.302	−0.015	−0.142	0.098	−0.334	0.049	−0.138	0.098	−0.330	0.053
Partner smoke	0.532	0.203	0.135	0.934	0.006	0.164	−0.313	0.325	0.011	0.218	−0.418	0.440	0.008	0.215	−0.412	0.431
Cigarettes/day	0.300	0.040	0.222	0.378	0.064	0.032	0.000	0.127	0.073	0.044	−0.012	0.158	0.076	0.044	−0.010	0.162
Intercept_Negative Affect																
Intercept	2.338	0.081	2.179	2.500	2.227	0.069	2.090	2.365	2.294	0.094	2.110	2.480	2.279	0.093	2.097	2.462
Age	−0.039	0.030	−0.099	0.021	−0.051	0.025	−0.100	−0.001	−0.062	0.034	−0.129	0.003	−0.058	0.033	−0.122	0.008
Ethnicity_African	−0.243	0.070	−0.380	−0.105	−0.236	0.059	−0.352	−0.121	−0.218	0.079	−0.370	−0.062	−0.219	0.079	−0.373	−0.065
Ethnicity_Hispanic	−0.308	0.074	−0.454	−0.165	−0.093	0.063	−0.217	0.030	−0.099	0.085	−0.265	0.068	−0.109	0.084	−0.273	0.057
Ethnicity_other	1.188	0.199	0.800	1.581	0.026	0.168	−0.308	0.357	−0.192	0.222	−0.630	0.248	−0.213	0.221	−0.647	0.223
Partner live together	−0.626	0.069	−0.763	−0.492	−0.125	0.058	−0.237	−0.011	−0.132	0.077	−0.283	0.021	−0.130	0.077	−0.282	0.019
Partner smoke	0.660	0.154	0.354	0.962	0.000	0.128	−0.251	0.253	−0.008	0.171	−0.343	0.334	−0.009	0.172	−0.341	0.327
Urge_(t-1) -> Urge_(t)																
Intercept	0.099	0.021	0.059	0.140	0.174	0.021	0.131	0.214	0.303	0.039	0.226	0.378	0.218	0.037	0.146	0.289
Age	−0.008	0.007	−0.023	0.006	−0.017	0.008	−0.033	−0.002	0.017	0.015	−0.012	0.045	0.021	0.013	−0.005	0.048
Ethnicity_African	−0.034	0.018	−0.068	0.000	0.040	0.018	0.006	0.074	−0.020	0.034	−0.088	0.047	0.001	0.031	−0.061	0.062
Ethnicity_other	−0.040	0.054	−0.146	0.063	−0.126	0.054	−0.233	−0.020	−0.224	0.116	−0.448	0.005	−0.076	0.100	−0.274	0.121
Education level	−0.004	0.015	−0.033	0.024	−0.012	0.015	−0.042	0.018	−0.057	0.028	−0.111	−0.002	−0.045	0.027	−0.098	0.006
Partner live together	0.036	0.017	0.002	0.070	−0.015	0.018	−0.050	0.020	0.002	0.034	−0.064	0.068	0.002	0.031	−0.060	0.062
Partner smoke	0.010	0.039	−0.065	0.088	−0.120	0.038	−0.195	−0.043	−0.029	0.081	−0.191	0.124	−0.053	0.069	−0.188	0.081
Cigarettes per day	−0.009	0.007	−0.024	0.006	0.024	0.008	0.009	0.039	0.051	0.014	0.023	0.079	0.036	0.014	0.010	0.063
Partner live together × Partner smoke	−0.054	0.044	−0.144	0.029	0.118	0.044	0.030	0.203	0.011	0.091	−0.163	0.193	0.041	0.078	−0.114	0.196
Negative Affect_(t-1) - > Negative Affect_(t)																
Intercept	0.052	0.021	0.010	0.095	0.191	0.023	0.146	0.236	0.261	0.040	0.183	0.339	0.198	0.036	0.126	0.268
Age	−0.016	0.008	−0.032	−0.001	−0.008	0.009	−0.025	0.009	0.016	0.015	−0.013	0.045	0.008	0.014	−0.019	0.034
Gender	0.001	0.015	−0.028	0.030	−0.059	0.016	−0.091	−0.026	−0.032	0.029	−0.090	0.025	−0.024	0.026	−0.075	0.026
Ethnicity_African	−0.001	0.018	−0.037	0.034	−0.039	0.019	−0.076	−0.002	−0.079	0.035	−0.148	−0.011	−0.053	0.031	−0.112	0.008
Ethnicity_Hispanic	−0.001	0.020	−0.039	0.037	−0.049	0.021	−0.091	−0.008	0.004	0.037	−0.068	0.076	0.005	0.034	−0.062	0.071
Ethnicity_other	−0.134	0.056	−0.244	−0.024	−0.056	0.061	−0.175	0.063	−0.174	0.145	−0.478	0.095	−0.243	0.102	−0.443	−0.044
Education level	0.030	0.015	−0.001	0.059	0.040	0.016	0.008	0.073	0.052	0.029	−0.005	0.108	0.043	0.026	−0.008	0.095
Partner live together	−0.056	0.018	−0.091	−0.020	−0.029	0.019	−0.066	0.010	−0.032	0.034	−0.099	0.035	−0.026	0.031	−0.088	0.034
Partner smoke	−0.101	0.043	−0.186	−0.016	−0.051	0.043	−0.136	0.033	−0.004	0.082	−0.172	0.155	0.008	0.068	−0.126	0.141
Partner live together × Partner smoke	0.135	0.049	0.038	0.232	0.049	0.049	−0.048	0.146	0.052	0.091	−0.128	0.235	0.024	0.078	−0.128	0.177
Negative Affect_(t-1) -> Urge_(t)																
Age	−0.003	0.007	−0.017	0.010	0.015	0.007	0.001	0.029	0.001	0.014	−0.027	0.028	0.003	0.010	−0.017	0.023
Ethnicity_other	0.106	0.049	0.010	0.203	0.011	0.051	−0.089	0.110	−0.065	0.117	−0.300	0.160	−0.047	0.080	−0.205	0.109
Partner live together	−0.072	0.015	−0.103	−0.042	0.026	0.016	−0.006	0.058	−0.034	0.030	−0.094	0.025	−0.030	0.024	−0.076	0.017
Partner smoke	−0.072	0.036	−0.140	−0.001	0.040	0.035	−0.027	0.108	0.042	0.070	−0.093	0.181	−0.003	0.051	−0.105	0.097
Partner live together × Partner smoke	0.109	0.041	0.027	0.186	−0.067	0.040	−0.145	0.009	−0.023	0.078	−0.180	0.127	0.014	0.058	−0.100	0.132
Urge_(t-1) -> Negative Affect_(t)																
Intercept	0.038	0.017	0.004	0.072	0.025	0.015	0.000	0.060	0.090	0.031	0.026	0.147	0.058	0.024	0.010	0.103
Ethnicity_African	−0.014	0.015	−0.044	0.015	0.043	0.013	0.014	0.067	0.027	0.027	−0.026	0.081	0.011	0.020	−0.025	0.054
Education level	−0.007	0.012	−0.032	0.017	−0.050	0.012	−0.072	−0.026	−0.031	0.022	−0.073	0.013	−0.014	0.018	−0.047	0.020
Partner live together	−0.018	0.015	−0.047	0.011	0.048	0.014	0.021	0.075	0.007	0.027	−0.046	0.059	−0.013	0.022	−0.058	0.028
Partner smoke	0.011	0.037	−0.064	0.082	−0.064	0.028	−0.119	−0.004	−0.044	0.069	−0.184	0.090	−0.003	0.049	−0.090	0.103
Random Effects																
Urge_intercept	0.690	0.027	0.640	0.744	0.550	0.022	0.508	0.595	0.725	0.030	0.669	0.786	0.715	0.029	0.660	0.775
Negative Affect_intercept	0.516	0.021	0.477	0.557	0.424	0.017	0.391	0.459	0.557	0.024	0.512	0.606	0.551	0.024	0.506	0.600
Urge_(t-1) -> Urge_(t)	0.075	0.008	0.060	0.091	0.078	0.008	0.064	0.093	0.170	0.013	0.146	0.196	0.171	0.012	0.150	0.195
Negative Affect_(t-1) -> Negative Affect_(t)	0.083	0.008	0.068	0.098	0.098	0.008	0.083	0.113	0.183	0.013	0.158	0.209	0.174	0.012	0.152	0.198
Negative Affect_(t-1) -> Urge_(t)	0.048	0.012	0.010	0.067	0.054	0.009	0.035	0.071	0.140	0.014	0.114	0.167	0.091	0.013	0.066	0.116
Urge_(t-1) -> Negative Affect_(t)	0.033	0.014	0.003	0.057	0.018	0.011	0.002	0.041	0.088	0.016	0.055	0.117	0.042	0.019	0.007	0.077
Time Varying Covariates Effects																
Lapse_(t-1) -> Urge_(t)	0.008	0.004	0.000	0.015	0.018	0.008	0.003	0.033	−0.040	0.008	−0.056	−0.024	−0.021	0.009	−0.039	−0.002
Lapse_(t-1) -> Negative Affect_(t)	0.011	0.004	0.003	0.019	0.018	0.008	0.003	0.033	0.025	0.010	0.006	0.044	0.011	0.009	−0.007	0.029
Lapse_(t) -> Negative Affect_(t)	−0.017	0.004	−0.025	−0.009	0.023	0.008	0.007	0.038	0.039	0.014	0.010	0.063	0.037	0.010	0.018	0.056
Cig availability_(t) -> Urge_(t)	0.130	0.005	0.120	0.140	0.075	0.004	0.068	0.083	0.038	0.007	0.024	0.052	0.043	0.007	0.029	0.057
Cig availability_(t) -> Negative Affect_(t)	0.009	0.005	−0.001	0.019	0.061	0.004	0.054	0.069	0.016	0.007	0.003	0.029	0.021	0.007	0.007	0.035

Part of the parameter estimates were presented here for purpose of comparing missing data handling approaches. Complete results were summarised in Supplemental Table S1.

Comparing parameter estimation results using different missing data handling approaches, we observed consistent findings in some but not all of the parameters. Cigarette availability was found to be positively associated with negative affect, indicating participants who had cigarettes available to them in a certain time period experienced more negative affect in the same time period. The intercept estimates for random-intercept for both urge and negative affect, as well as the AR coefficients of the two DVs, were all credibly different from zero regardless of missing data handling approaches. In addition, the mean level of negative affect across days (i.e., random-intercept for negative affect) was estimated to be lower for African Americans than for than Whites.

When multiple imputation approaches were used to handle the missingness—whether it was single or multilevel MI—more credible fixed effects (i.e., effects with credible intervals not covering zero) were observed and the magnitudes of most of the random-effect estimates were smaller than those observed utilizing BFIML and LD. This highlighted the sensitivity of the modeling results to the presence of potential extreme values of the DVs. The participants were trained to self-initiate “urge assessment” in response to high levels of urge, which are generally accompanied by high levels of negative affect. Thus, by design, we would expect higher levels of urge and negative affect in self-initiated assessments. Such self-initiated surveys are one example of event-contingent designs. The high levels of urge collected during the self-initiated surveys are representative of the participants’ underlying states. Using that information in the imputation procedure will inform urge/negative affect levels of missed observations adjacent to the lapse. Incorporating self-initiated surveys in the imputation process was one possible way to enable us to utilize both types of assessments in examining participants’ recovery pathways, but this may not be the only way. In addition, consistent with previously reported results, VAR parameter estimates were generally smaller in magnitudes in the LD than other approaches. This was expected since LD altered the time intervals between successive observations, leading to violation of the assumption of equally spaced time intervals in the VAR models and consequently, biased estimates especially for the VAR parameters.

Comparing results from multi-level MI to those from single-level MI, more credible effects were observed for fixed effects of random-intercepts and some of the random-VAR parameters with multilevel MI. For instance, lower intercepts of urge (i.e., lower baseline level of urge) were observed among participants who were African American, lived with partner, especially partner who did not smoke, and who smoked fewer cigarettes per day. In terms of baseline negative affect, participants who were African American and Hispanic, relative to White and other ethnic groups, lived with partner, partner who did not smoke, were characterized by lower baseline negative affect.

We observed interesting interaction effects of whether participants lived with their partners and whether their partners smoked on the dynamic parameters (i.e., AR and CR parameters) for urge and negative affect. For instance, comparing with participants living alone and those living with smoking partner, participants who lived with non-smoking partners had the lowest baseline levels for both urge and negative affect. Also, the negative CR coefficient for previous time negative affect on urge for participants living with non-smoking partners led to lower levels of urge for this group as compared to the other two groups. However, all partner effects observed in this analysis were small in magnitude and future studies shall examine these effects and see if they replicate. The positive CR effect of previous urge on negative affect, indicating that higher than usual urge at a previous time block was associated with higher than usual negative affect at the current time point. In addition, we also observed a credible negative effect for age on the AR coefficient of negative affect. This age-related effect indicated that older participants had lower AR values and thus quicker return to their baseline negative affect level following any deviations from it.

Lapse at the current and previous blocks (t and t−1, respectively) were included as covariates in the VAR model. One effect found to be credibly different from zero across most approaches (except for LD) was that higher previous lapse was credibly associated with higher negative affect at time t. The concurrent association between lapse and negative affect within the same time block was also found to be positive under most approaches but not the full MI ML approach. In a similar vein, the lagged association from previous lapse on current urge also showed inconsistent variations in magnitudes and signs across approaches. Overall, this suggested that complex associations might exist between lapse and urge within and across time blocks. In the current study, many lapse observations were reported retrospectively and time of the lapse were adjusted based on the participants’ report based on available information. In addition, we aggregated EMA responses into six-hour blocks. Those uncertainty about temporal ordering of lapse, urge, and negative affect might lead to missed opportunities to detect these relations at a more nuanced level.

In the current application, the estimation results using BFIML approach for many parameters were different from the MI approaches. This was mainly due to the fact that with BFIML model, only data from random EMAs were modeled. In other words, we did not incorporate information from self-initiated EMAs as we did with MI approaches. This may lead to underestimated levels of urge and negative effect. Future research shall investigate methods to include auxiliary variables with BFIML approach in MVAR models, especially when we expect non-ignorable missing data.

To summarize, comparing results from different missing data handling approaches, we observed more credible effects when we incorporated information from self-initiated EMAs and proper MI procedures (i.e., multilevel MI) were used. Credible between-person differences were observed with both baseline levels and AR CR parameters for the two DVs. Among the person-specific covariates we tested in this model, we found that age, cigarettes per day, partner smoking status, and ethnicity had credible effects on either baseline level or AR/CR parameters of the MVAR model. In addition, at the within-person level, cigarette availability was associated with higher levels of urge relative to personal baselines; lapse was associated with higher negative affect level at the following time block but lower negative affect level during the same time block.

## 5. Discussion

With the growing interest in studying between-person differences in the dynamics of behavioral processes, as well as the widespread availability of portable and wearable devices that allow for intensive data collection, we have seen more applications of MVAR models in behavioral and social sciences in recent years. However, practical issues also arise in the implementation of this rather complex model, missing observation treatment being one of them. Available software that supports the fitting of MVAR models often resorts to heuristic methods of handling missing data. For instance, the R-package mgm (62) only uses complete cases for modeling time-varying VAR models (63). Mplus adopts a Bayesian estimation approach based on MCMC algorithm via the Gibbs sampler for dynamic SEM. Missing data in dependent variables were treated as parameters (22). If there are missing covariates, they need to be modeled the same way as other dependent variables with appropriate distributional assumptions (64). This approach is similar as the BFIML approach we described in our simulation. Other sophisticated approaches can and have been implemented for handling missingness involving longitudinal panel data (65, 66). Building on existing research on multilevel MI and MI implementations with dynamic models, this paper extends our knowledge of missing data treatments for MVAR models and illustrates the utility of this approach with EMA study data.

It is well recognized in the cross-sectional literature that multilevel structures need to be accounted for in modeling clustered data. Similarly, multilevel imputation, which accommodates the clustering of data in the imputation procedure, is necessary to avoid inaccurate estimation of model parameters. Studies comparing multilevel imputation with single-level imputation for cross-sectional multilevel regression models demonstrated that when single-level imputation was used, the within-level regression coefficients tended to be overestimated, whereas the between-level regression coefficient was underestimated (40). In addition, over-estimation of parameter SE for the within-level parameters, and under-estimation of parameter SE for the between-level parameters are expected (14). These biases in estimates tend to lead to low coverage, though power may not be affected. In contrast, our simulation study revealed, in the context of the MVAR model and data that were MAR, more complex patterns of biases across different types of parameters. For the within-level model, dynamic parameters, including AR and CR parameters, were all under-estimated, but process noise variances and covariances were over-estimated. Similar to cross-sectional models, SEs for the within-level parameters were over-estimated for dynamic parameters, but only when the number of per-cluster observations (i.e., the number of time points in the present simulation studies) was small. For process noise variance-covariance parameters, underestimation in SEs was noted instead, especially when the per-cluster sample size was large and ICC was high. Interestingly, the estimation of parameters related to the time-varying COVs in the within-level model was robust regardless of imputation methods. This was mainly due to the fact that the COVs involved in the simulation study did not have a clustering structure, and were designed to comprise only fixed effects.

**Figure 5:**
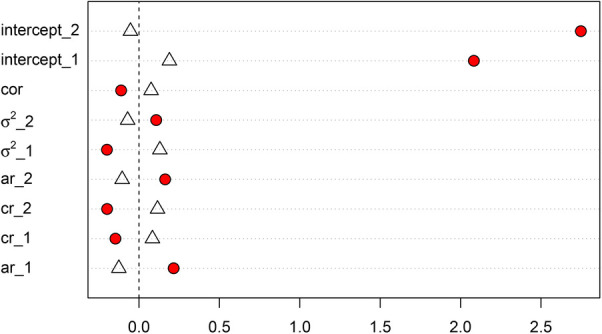
Comparison of biases of selected model parameters estimates with (1) multilevel MI for intensive longitudinal data, and (2) conventional multilevel MI. Biases of model parameter estimates with multilevel MI for intensive longitudinal data are indicated by triangles. Biases of model parameter estimates with conventional multilevel MI are indicated by red circles.

One distinct feature of implementing multi-level MI to multi-subject multivariate intensive longitudinal data is that we included lagged variables in the imputation model. This is because with time series data, we assume observations at one time point will have an impact on the observations at the next time point, hence previous time point observations may help predict missing observtion at the current time point, if there is any. We illustrated the difference of multi-level MI for intensive longitudinal data (i.e. including lagged variables) and conventional multi-level MI for cross sectional data (i.e. without lagged variables) using one of the simulated dataset in the simulation study. Biases of model parameter estimates with the two different approaches were plotted in Figure 5. For fixed effects of the random intercepts and all dynamic parameters, including AR, CR, and process noises parameters, multilvel MI for intensive longitudinal data had smaller biases when compared with conventional multilevel MI without lagged variables in the imputation model. We expect that including lagged variables is helpful in multilevel MI for intensive longitudinal data. This result is consistent with prior simulation with single-level MI for intensive longitudinal data (13). The differences of biases of other model parameters, such as time varying covariate effects, are smaller.

In addition, our results also underscored the importance of integrating information from the self-initiated (slip) EMAs into the modeling process in tobacco control studies. In the present study, we found that inclusion of information from the self-initiated EMAs helped provide imputed values of urge and negative affect that would otherwise constitute a source of MNAR data. By design, participants in the present study were asked to provide self-initiated EMAs when their urge, and by association, their negative affect, were the highest. Thus, we would expect higher levels of urge and negative affect in self-initiated assessments. Moreover, because participants choose when to initiate an assessment, self-initiated assessments will be plagued by self-selection bias and prior research has suggested that they do not capture the most extreme moments. In addition, there are no assessments where participants are trained to report low levels of urge for example. As such, the limitations of self-initiated assessments in the current study need to be acknowledged in that they are biased by self-selection and are not representative of the full range of experience (e.g., both high and low levels). In the current study, including self-initiated EMAs in the multilevel MI approach yielded more credible fixed effects, such as effects of ethnicity, partner status, partner smoking status, and cigarettes per day on baseline levels of urge and negative affect. These fixed effects help inform future developments of more targeted interventions for participants from diverse demographic backgrounds. In addition, we also found substantial differences in intercepts (i.e., baseline values) for both urge and negative effect for different ethnic groups. Closer examination of the reasons/mechanisms for these racial differences in future studies are warranted.

The MVAR model applied in this study is a discrete-time model looking at within person lag/cross-lag effects among variables overtime, and between person differences in these effects. Models that include contemporaneous effects between change processes, such as structural vector autoregressive models (67, 68) and other continuous-time extensions (69, 70) may be needed to better capture the interdependence among these changes processes at the most strategic time scales. Continuous-time models also do not require data binning as a data processing procedure, which may lead to more accurate estimation results. In addition, incorporating information from both random and self-initiated surveys, longitudinal mediation models and Bayesian structural equation models, which are special case of MVAR, would be helpful to test specific mediational hypotheses (71–74).

Further expansions of the MVAR models investigated in this paper to include observed measures of mixed measurement and distributional characteristics (e.g., categorical and other non-Gaussian variables; (75)) and simultaneous modeling of change processes and missing data mechanisms (49, 76) are also critical to help hasten our understanding of individuals’ dynamics in tobacco control studies. In addition, the MVAR model we applied in this study focused on modeling the intra-individual variability overtime and the inter-individual differences in those variability. If the research interest in studying how the relationships of risk factors and lapses change overtime, researchers may consider using models specifically looking at time varying effects, such as Time-Varying Effect Model (TVEM; (57, 77)). In the current empiricial data illustration, we focused on the effect of time-varying covariates on the DVs at the within-person level. Future research shall decompose time-varying covariates into within- and between-person effects and explore the effects at both within- and between-person levels.

The simulation and empirical studies in the present article are characterized by several other limitations that might be circumvented with improved designs in future studies. In the simulation study, we only considered MAR missing data. Ji et al.’s (13) simulation study found that with a single-level VAR model, MI procedures performed reasonably well with non-ignorable missingness. Therefore, future simulations may explore the performance of multilevel MI when data are missing not at random. The simulation in this study examined how sample size may have an influence on model performance, but did not test the impact of percentage of missing data and number of imputations needed with different amount of missing information. It would also be important to examine how magnitude of AR and CR effects may have an impact on the estimation when missing data exists and how different multilevel MI tools perform different with different AR and CR. More extensive simulation studies are needed to explore ways to determine the optimal number of imputations needed for multilevel MI under different scale of missingness. Lastly, in this study, we did not consider situations when participants differ in their missing data model. It would be important to evaluate the performance of the multilevel MI approach when multilevel structure exist not only with empirical study model, but also with the missing data model. Future research shall also explore and compare group-based multi-level MI with imputing each persons data with different timepoint and number of participants combinations.

## 6. Conclusion

This study demonstrated the importance of accounting for multilevel structure in the MI procedures for ILD when fitting MVAR models using simulated data. This missing data handling procedure can be applied in EMA studies, which are helpful in improving the efficacy of interventions. Without proper handling of the clustered missingness, there may be underestimation of the association of the variables of interest over time, which could lead to underestimation of the participant’s resistance to change. With empirical EMA data from a tobacco-cessation study, we illustrated a novel way of incorporating self-initiated EMAs to inform missing observations in the variables of interest, which are obtained using random EMAs. With this approach, we reduced the occurrence of NMAR and found more effects of substantive interests. Results of the current study may inform future tobacco cessation intervention tailored to population of varying demographic backgrounds.

## Data Availability

The original contributions presented in the study are included in the article, further inquiries can be directed to the corresponding author/s.

## Footnotes

^1^In a separate simulation study, we compared estimation results using the above described Bayesian posterior-distribution-based pooling methods with the Rubin’s method (25). Results showed the two approaches provided comparable results in the simulation conditions tested in this manuscript.
